# Do Full-Length Femoral Radiographs Influence Fixation Method in Proximal Femur Fractures in Patients With a Co-existent History of Malignancy?

**DOI:** 10.7759/cureus.101439

**Published:** 2026-01-13

**Authors:** Thomas MacKinnon, Edward Matthews, Rajarshi Bhattacharya

**Affiliations:** 1 Trauma and Orthopaedics, Imperial College Healthcare NHS Trust, London, GBR

**Keywords:** extracapsular hip fracture, hip and proximal femur trauma, malignancy, neck of femur fracture, xray

## Abstract

Purpose

In patients admitted with extracapsular proximal femur fractures (PFFs) and a co-existent history of malignancy, pre-operative full-length femoral radiographs (FLF-XRs) should be obtained to exclude bony metastases and select the appropriate fixation method. It is unclear whether such radiographs influence the surgeon’s chosen fixation method, even when no metastases are identified. Our primary aim was to identify whether FLF-XRs influenced the fixation method in this cohort.

Methods

We performed a retrospective analysis of all patients with PFFs who underwent surgical fixation at a major London teaching hospital over a three-year period (2018-20) using a search of electronic patient records. Data including history of any malignancy, FLF-XR status, fixation method (intramedullary nail (IMN) or sliding hip screw (SHS)), and one-year morbidity and mortality were collected.

Results

Our results showed that of the 306 extracapsular PFFs admitted during this time period, 23% (n = 69) had a history of malignancy, and of these, 55% (n = 38) had undergone FLF-XRs. Breast cancer was the most common primary source of co-existing malignancy. After excluding all subtrochanteric fractures (which structurally necessitate IMN fixation), there was no significant difference in fixation method resulting from the presence (or absence) of FLF-XRs in neither the co-existent malignancy PFF group (p = 0.09) nor the without malignancy PFF group (p = 0.84). Within the one-year post-operative follow-up period, none of the PFF patients with co-existent malignancy subsequently re-presented to the study hospital with complications relating to their fracture or surgical fixation. The one-year mortality rate was 28% (n = 19) amongst this group.

Conclusion

Excluding fracture patterns that necessitate a specific fixation method (subtrochanteric PFFs), the presence of FLF-XRs does not influence the chosen method of fixation (between IMN and SHS). We concur with existing literature that pre-operative FLF-XRs in PFF patients with co-existent malignancy are a low-value investigation and should not delay surgery beyond 36 hours from admission.

## Introduction

Proximal femur fractures (PFFs), or neck of femur fractures, represent the commonest reason for admission to acute orthopaedic wards [1]. Most commonly resulting from low-energy injury mechanisms, such as falls from standing, PFFs predominantly affect older, frail, osteoporotic patients often with co-morbid medical conditions. As the UK population continues to age, it is predicted that the incidence of PFFs will continue to rise - with up to 100,000 cases per year projected by 2033 in England alone [2].

Another anticipated consequence of an improved life expectancy is an increase in the incidence of malignancy or those living with previously treated cancer. Earlier diagnoses resulting from population-wide screening, for example, the National Health Service (NHS) breast cancer screening programme in the UK [3,4], along with ever-improving oncological therapeutics, are leading to more patients living with or having been treated for cancer. In any patient with a history of cancer presenting with a PFF, the possibility of a pathological fracture or malignant bony deposits distal to the fracture must be considered when planning orthopaedic surgery.

In the United Kingdom, it is common practice in the pre-operative investigation of any patient presenting with a PFF and co-existent history of malignancy (PFF-CM) to obtain full-length femoral (FLF) anteroposterior and lateral view radiographs, in addition to standard pelvic and hip radiographs - a guideline set out by the British Orthopaedic Association (BOA) first in 2015 [5] and again in the 2022 updated BOA guideline [6]. These radiographs serve to help identify any metastatic bony disease distal to the fracture site and thus reduce the risk of subprosthetic fracture by allowing the surgeon to select the most appropriate implant - however, there is limited evidence to justify this practice. The BOA guideline for the care of the older or frail orthopaedic trauma patient also recommends surgery to allow for full weight-bearing activities within 36 hours of admission, in line with current hip fracture care [7].

Not all PFF-CM patients have FLF radiographs (FLF-XRs) performed by the time of the trauma meeting. Obtaining these radiographs can result in a delay to surgery beyond the 36-hour window from admission recommended by the BOA, if there is clinical suspicion of a cancer history. It has been observed that for patients with extracapsular PFF-CMs, requiring fixation with either a sliding hip screw (SHS) or intramedullary nail (IMN) system, the presence of FLF-XRs at the trauma meeting could influence the choice of fixation method - even when no metastatic pathology of the femur was demonstrated.

The purpose of our study was therefore to review all PFF patients with a co-existent history of malignancy over a three-year period to determine exactly what proportion of extracapsular PFF patients underwent FLF-XRs, whether these radiographs identified metastatic bony pathology, and what fixation method was opted for - in order to establish whether the presence of FLF-XRs influenced the fixation method.

## Materials and methods

A retrospective observational study was carried out at St Mary's Hospital - Imperial College NHS Trust, a major trauma centre in London, analysing all adult (aged over 16 years) PFF patients admitted under the care of the trauma and orthopaedic surgery team over a three-year period (January 1, 2018, to December 31, 2020). Patients with a confirmed history of malignancy were included in the PFF-CM group, regardless of whether metastatic disease had been previously diagnosed by other imaging modalities or clinical records. Patients with intracapsular fractures (requiring arthroplasty), high-energy mechanisms (energy greater than falls from standing), or periprosthetic fractures were excluded. For missing data, all available information from the hospital electronic records and the Summary Care Record was used; no data imputation was performed.

All extracapsular PFF patients who underwent surgical fixation during this period were identified from the local hospital PFF database. For each patient, data collection points including patient demographics, documented history of cancer, fracture configuration (intertrochanteric vs. subtrochanteric), and orthopaedic fixation method used were extracted from the electronic record. This included a review of the admission trauma meeting discussion records and operation notes. Data collection of one year post-operative complications, including revision surgery, and mortality was also accessed using the hospital electronic notes, as well as from the incorporated ‘Summary Care Record’, an electronic medical record-keeping network from surrounding local hospitals (for example, to identify if a patient had presented to another hospital or their general practitioner with a post-operative complication). The hospital electronic radiology imaging software was used to identify the fracture configuration (intertrochanteric, subtrochanteric) as well as to record the pre-operative FLF-XR status. Data were collected for all patients, including both PFF-CMs and PFF patients with no co-existent history of malignancy (PFF-NCM), to provide a control group. Data collection was performed by the study's primary team members. Fisher’s exact test was used for statistical analysis. Ethical approval for this local service evaluation was acquired via the local audit and research department.

## Results

**Figure 1:**
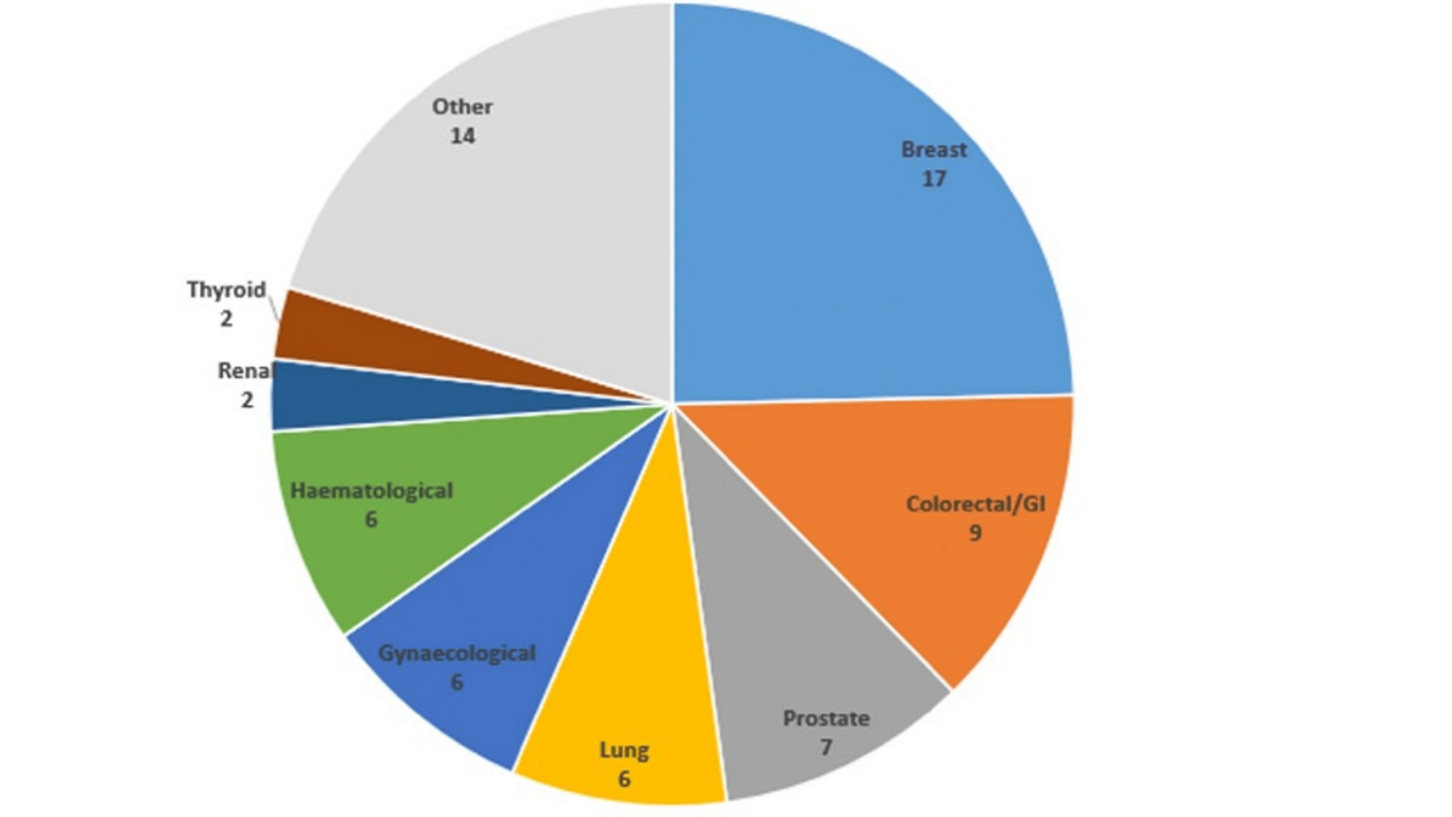
Chart showing breakdown of origin of primary cancer types in the study group. GI: gastrointestinal

Over the three-year period, a total of 650 PFFs were identified, 306 of which (47%) were extracapsular fractures requiring fixation (mean age: 83, F:M ratio 3:1, with similar demographic breakdown in subsequent groups). Of these, 22.4% (n = 69) of patients had a co-existent history of malignancy. The most frequently observed primary malignancies with bone metastases were breast cancer (n = 17), gastrointestinal or colorectal cancers (n = 9), and prostate cancer (n = 7) (Figure 1).

One PFF-CM patient had metastatic bony disease identified on FLF-XR (2.6%), consisting of one mid-femoral metastatic deposit of prostatic origin. Within the one-year post-operative follow-up period, none of the PFF-CM patients subsequently re-presented to the study hospital with complications relating to their fracture in the form of subprosthetic fracture or unsupported distal disease. The one-year mortality rate was 28% (n = 19) in the PFF-CM group and 21% (n = 50) in the PFF-NCM group.

**Figure 2:**
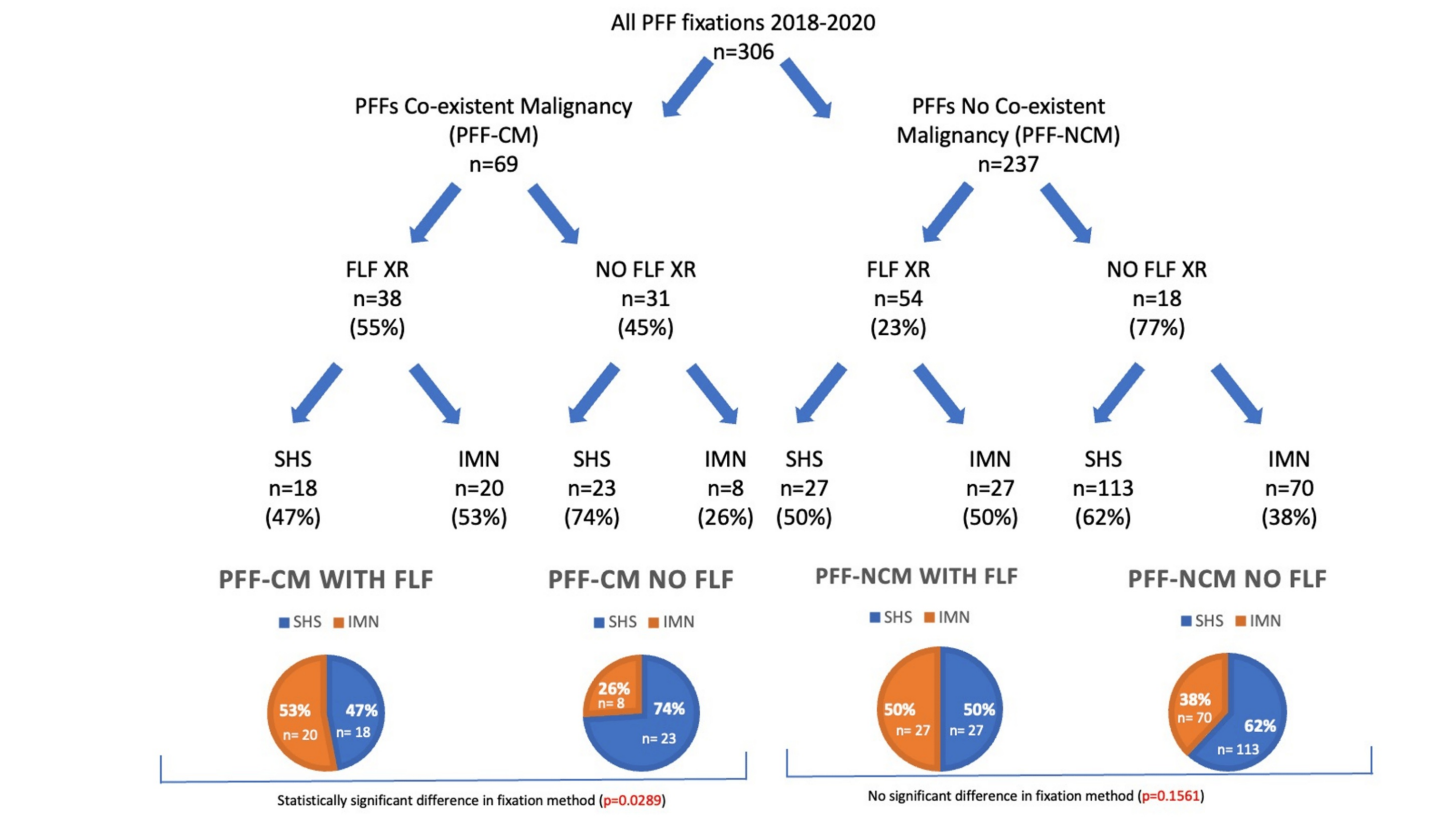
Flow chart showing breakdown of all extracapsular PFF fixations into co-existing malignancy history status (PFF-CM/PFF-NCM), FLF-XR status, and surgical fixation method used (SHS/IMN). All fracture configurations were included in this statistical analysis (including trochanteric and subtrochanteric) and resulted in a positive FLF-XR status influencing fixation method amongst the PFF-CM group towards IMN to a statistically significant degree (0.0289) when compared to the PFF-NCM group (p = 0.1561). PFF-CM: proximal femur fracture with history of co-existent malignancy; PFF-NCM: proximal femur fracture with no history of co-existent malignancy; FLF-XR: full-length femoral radiograph; IMN: intramedullary nail; SHS: sliding hip screw; PFF: proximal femur fracture

Of the 69 extracapsular PFF-CM patients admitted during the study period (Figure 2), pre-operative FLF-XRs were obtained for 38 of these (55%). Of this group, IMN and SHS fixation rates were similar (20 (53%) IMN vs. 18 (47%) SHS). By contrast, of the 31 PFF-CMs for whom FLF-XRs were not obtained, SHS fixation was nearly three times more likely to be performed compared to IMN fixation (23 (74%) SHS vs. 8 (26%) IMN). Accounting for all fracture configurations (trochanteric and subtrochanteric), this difference in fixation method in the FLF and non-FLF groups was statistically significant (p = 0.0289).

As a control, the same analysis was performed on the 237 remaining extracapsular PFF-NCM. Fifty-four (23%) of these underwent pre-operative FLF-XRs, and of these, equal numbers of SHS and IMN fixations were performed (27). Amongst the group for whom FLF-XRs were not available pre-operatively, more SHS fixations were performed (113 (62%) SHS vs. 70 (38%) IMN); however, this difference between FLF and non-FLF-XR groups did not reach statistical significance (p = 0.1561).

**Figure 3:**
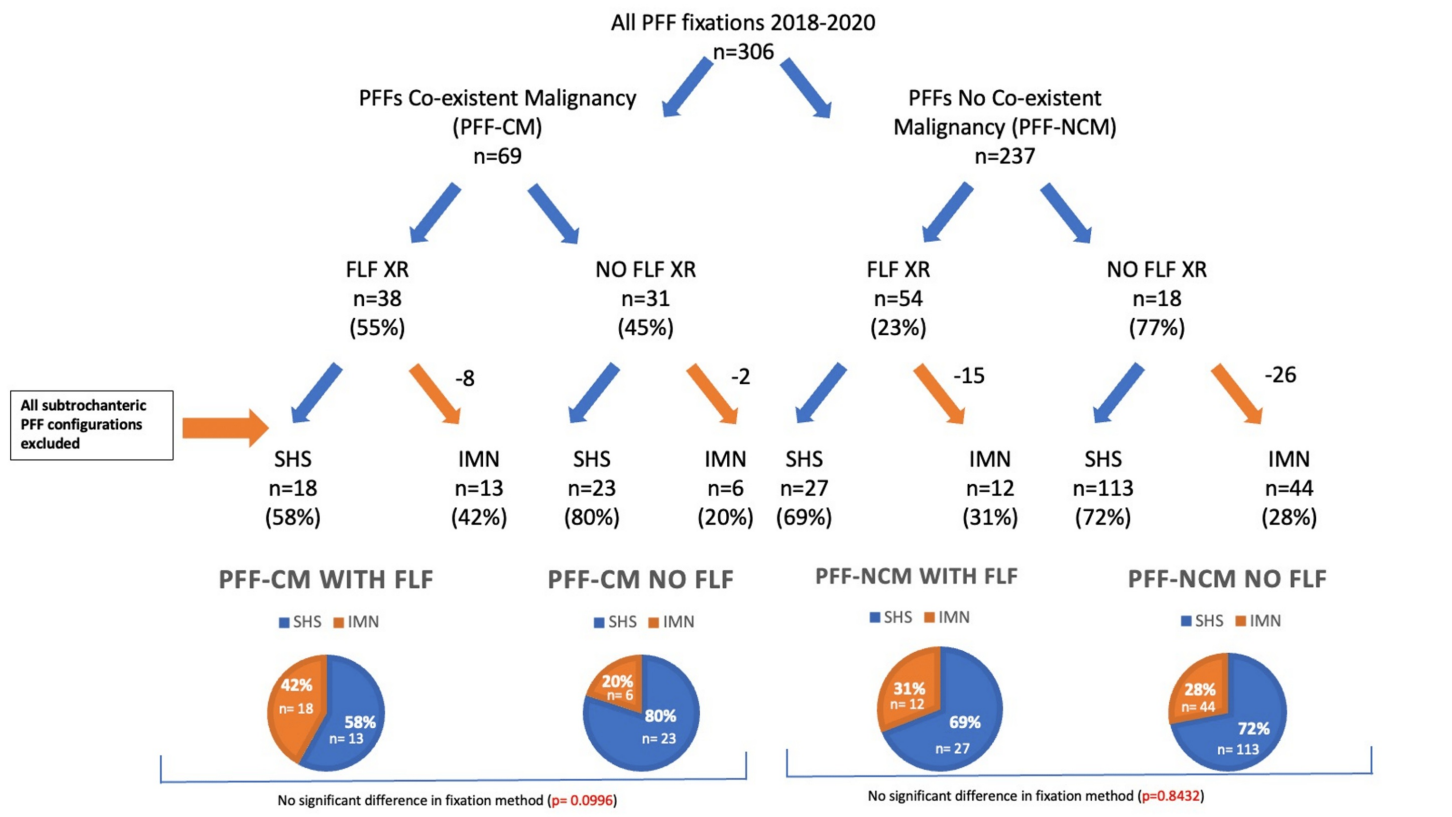
Revised flow chart once again showing breakdown of all extracapsular PFF fixations into co-existing malignancy history status, FLF-XR status, and fixation method. In this analysis, all subtrochanteric fractures were excluded (orange arrows), resulting in no statistically significant difference in fixation method secondary to FLF-XR status in either PFF-CM or PFF-NCM groups. PFF-CM: proximal femur fracture with history of co-existing malignancy; PFF-NCM: proximal femur fracture with no history of co-existing malignancy; FLF-XR: full-length femoral radiograph; IMN: intramedullary nail; SHS: sliding hip screw; PFF: proximal femur fracture

Importantly, given that PFFs with a subtrochanteric fracture configuration are not compatible with SHS fixation and inherently require IMN fixation, the patients with subtrochanteric fracture patterns were identified (Figure 3). These included 10 subtrochanteric fractures amongst the PFF-CM group (of which eight had FLF-XRs), and 41 in the PFF-NCM group (of which 15 had FLF-XRs). After excluding all such cases treated with IMN fixation and repeating the analysis, there was no statistically significant difference in fixation method secondary to FLF-XR status in either PFF-CM (p = 0.0996) or PFF-NCM (p = 0.8432) groups. 

## Discussion

In this study, 38 of 69 extracapsular PFF-CMs (55%) underwent FLF-XRs prior to undergoing surgical fixation. BOA guidelines require ‘the following investigations should be conducted... orthogonal radiographs of the whole bone’ when pre-operatively assessing a fracture in the context of co-existing malignancy [5]. At present, however, there are no comparable, contemporary studies in the literature evaluating adherence to these guidelines in other centres across the United Kingdom, nor indeed is there a clear evidence base to support this guideline. Whilst the adherence rate in PFF-CMs of just over half in this study is notable and we advocate for further large-scale audits to further assess the adherence rate nationally, the purpose of the present study is to assess whether FLF-XRs actually yield any significant change to surgical practice or patient outcomes in the PFF-CM demographic.

In our study, metastatic bony pathology was identified in one of 38 (2.6%) pre-operative FLF-XRs amongst PFF-CMs. The single positive FLF-XR finding in this study was a mid-femoral metastatic lesion secondary to a prostatic carcinoma, and the patient underwent dynamic hip screw fixation due to the surgeon preference. A similar retrospective study by O’Flaherty et al. analysed FLF-XRs of 133 PFF-CM patients over a two-year period and found no femoral pathology in any of the cases [8]. A further, larger study of 424 patients by Ghobrial et al. identified only seven patients (1.6%) with metastatic bony lesions on FLF-XRs, and surgical intervention was not altered in any of these cases as a result [9]. Both studies concluded that FLF-XRs were of limited value in the pre-operative evaluation of this demographic of patients. Their conclusions are shared by this study in the context of extracapsular PFF, and questions are raised as to why FLF-XRs are performed for PFF-CMs if surgical practice does not alter in response to an abnormal finding (specifically, IMN rather than SHS fixation to treat a distant femoral metastasis).

With limited evidence regarding the diagnostic value of FLF-XRs in PFF-CMs, one key point for discussion is that of avoiding delay to surgery. Within the UK NHS, the use of hip-fracture best practice tariffs (BPTs) is widespread [10]. These are financially incentivised targets for hospitals to promote high-quality, standardised, and cost-effective care for PFF patients. A critical component of the NHS hip-fracture BPT programme is that the ‘time to surgery’, from the point of diagnosis to surgery, must be less than 36 hours (as recommended by the BOA) [7]. Frail, elderly PFF patients with co-morbid medical conditions are at risk of developing complications such as chest infections and deep venous thromboses. Any delay beyond 36 hours has been shown to result in adverse patient outcomes, and all PFF patients should therefore, from the point of presentation to the emergency department to surgery, have undergone all necessary pre-operative orthogeriatric evaluations and investigations within this timeframe [11,12]. In the case of PFF-CMs, one such rate-limiting investigation is the FLF-XR, and difficulties obtaining these scans (for example, due to reduced weekend staffing levels) can breach ‘time to surgery’ beyond 36 hours. We advocate that in such instances, waiting for FLF-XRs should never delay a hip fracture fixation surgery.

In PFF-NCM patients, the presence (or absence) of pre-operative FLF-XRs would not be expected to influence the fixation method chosen by the surgeon. This is consistent with our findings, with no significant difference in fixation method identified between the FLF and non-FLF-XR groups. In the PFF-CM group, without stratifying extracapsular fractures into trochanteric and subtrochanteric configurations, the presence of FLF-XRs initially appears to sway the fixation method chosen by the surgeon away from SHS fixation (which predominates in the PFF-NCM group) towards the use of the IMN and to a statistically significant degree (p = 0.0289). However, given that subtrochanteric fractures are inherently limited to IMN fixation due to their instability, after removing these from the analysis, this significance is lost (p = 0.0996), and therefore, FLF-XRs did not influence the fixation method in either PFF-CM or PFF-NCM group.

Not all patients with bony metastases will go on to sustain a pathological fracture. Whilst historic work by Galasko [13] in breast cancer patients suggested that destructive lesions must exceed approximately 1 cm in diameter with substantial (>50%) bone loss to be visible on plain radiographs, more contemporary studies across multiple tumour types have reported similarly low sensitivity of pre-operative FLF-XRs in detecting metastatic deposits [8,9]. Taken together, these findings support the observation in our cohort that FLF-XRs rarely identify distal metastases and suggest that routine pre-operative imaging may have a limited impact on surgical decision-making, prompting consideration of alternative approaches such as intraoperative femoral screening. Whilst our study argues against routine pre-operative FLF-XRs in extracapsular PFF-CM patients, intraoperative screening of the femur may serve as a pragmatic adjunct instead of pre-operative imaging as a screening tool. This approach is already used in related contexts, such as screening the femoral neck in ipsilateral femoral shaft fractures [14]. Further studies are required to establish its feasibility, accuracy, and cost-effectiveness in this patient population.

A limitation of the present study is the possibility of selection bias due to reliance on local hospital electronic records for mortality and morbidity data collection. For example, under-reporting of morbidity data may have resulted from patients presenting with surgical complications to other, distant hospitals with no shared electronic records. Similarly, given that elderly hip fracture patients, especially those with cancer, are increasingly likely to receive hospice care, under-reporting of mortality data may have resulted. Reassuringly, our one-year mortality rate (in the PFF-CM group) of 28% is consistent with figures reported in the literature (ranging from 14.8% to 37% [15-17]).

We acknowledge that fixation decisions are influenced by multiple factors beyond pre-operative imaging, including intraoperative findings, fracture stability, patient frailty, and surgeon preference. Adjustment for all potential confounders was not feasible in this retrospective study. Additionally, all FLF-XRs were reviewed according to standard departmental protocols, and interpretation was performed as part of routine clinical care by attending surgeons and radiologists. The single patient with a metastatic lesion that did not alter fixation highlights the low clinical yield of FLF-XRs in this context. Whilst we cannot definitively determine why fixation was unchanged in this case, surgeon judgement, fracture configuration, and implant choice are likely contributory factors. Despite these limitations, our study provides contemporary evidence that routine pre-operative FLF imaging rarely impacts fixation decisions in extracapsular PFF patients with a history of malignancy.

## Conclusions

Accounting for and excluding all subtrochanteric fractures that inherently necessitate IMN fixation, FLF-XRs in extracapsular PFF-CM patients do not influence surgeons’ choice of fixation method between IMN and SHS implants. This study agrees with existing literature that pre-operative FLF-XRs in PFF-CM patients are a low-value investigation and we argue should not be the sole reason for any delays to surgery.

Further larger-scale studies are also required to establish current national practices on FLF-XR adherence in the PFF-CM demographic. We also challenge the accepted practice that FLF-XR should be a ‘blanket approach’ pre-operative requirement in PFF-CM patients; instead, we propose that the FLF-XR should be an adjunctive investigation at the discretion of the operating surgeon. Additionally, we propose that intraoperative screening of the whole femur to screen for metastasis may be a viable alternative to FLF-XRs; however, it is clear that further research to define and validate this would be required to ensure reproducibility of such a screening technique.
